# Autonomous trisomic rescue of Down syndrome cells

**DOI:** 10.1038/s41374-019-0230-0

**Published:** 2019-02-13

**Authors:** Momoko Inoue, Kazuhiro Kajiwara, Ayumi Yamaguchi, Tohru Kiyono, Osamu Samura, Hidenori Akutsu, Haruhiko Sago, Aikou Okamoto, Akihiro Umezawa

**Affiliations:** 1 Department of Reproductive Biology, National Center for Child Health and Development, Tokyo, 157-8535 Japan; 2 Department of Obstetrics and Gynecology, The Jikei University School of Medicine, Tokyo, 105-8471 Japan; 3 Division of Carcinogenesis and Cancer Prevention, Department of Cell Culture Technology, National Cancer Center Research Institute, Tokyo, 104-0045 Japan; 4 Department of Maternal-Fetal, Neonatal and Reproductive Medicine, National Center for Child Health and Development, Tokyo, 157-8535 Japan

**Keywords:** Induced pluripotent stem cells, Experimental models of disease

## Abstract

Down syndrome is the most frequent chromosomal abnormality among live-born infants. All Down syndrome patients have mental retardation and are prone to develop early onset Alzheimer’s disease. However, it has not yet been elucidated whether there is a correlation between the phenotype of Down syndrome and the extra chromosome 21. In this study, we continuously cultivated induced pluripotent stem cells (iPSCs) with chromosome 21 trisomy for more than 70 weeks, and serendipitously obtained revertant cells with normal chromosome 21 diploids from the trisomic cells during long-term cultivation. Repeated experiments revealed that this trisomy rescue was not due to mosaicism of chromosome 21 diploid cells and occurred at an extremely high frequency. We herewith report the spontaneous correction from chromosome 21 trisomy to disomy without genetic manipulation, chemical treatment or exposure to irradiation. The revertant diploid cells will possibly serve a reference for drug screening and a raw material of regenerative medicinal products for cell-based therapy.

## Abstract (web summary)

Continuously cultivated iPSCs with chromosome 21 trisomy spontaneously reverted to normal diploids. This trisomy rescue occurred without genetic manipulation, chemical treatment, or exposure to irradiation. The revertant cells can serve as a reference for drug screening and as raw materials for regenerative medicine and cell-based therapy.

## Introduction

Recently, attention to prenatal diagnosis is increasing due to the higher average age of pregnant women. Due to the availability of diagnostic techniques such as non-invasive prenatal genetic testing and improvement of imaging technology, congenital diseases including chromosomal abnormalities are possible to diagnose earlier than before [1, 2]. On the other hand, there are few genetic disorders in which early diagnosis contributes to the improvement of the prognosis of children. Down syndrome is the most frequent chromosomal abnormality among live-born infants. All Down syndrome patients have mental retardation and are prone to develop early onset Alzheimer’s disease. In addition, leukemia, cardiac malformation, hearing disorders, and vision disorders are also seen at a high rate. Hyperkeratosis of the skin is occasionally observed [3]. Ninety percent of Down syndrome cases are due to an extra copy of chromosome 21 and the remainder exhibit imbalanced translocation or mosaicism. Triplication of specific regions of chromosome 21, band 21q22, causes various physical and cognitive phenotypes of Down syndrome, and the causative genes include amyloid beta precursor protein (APP) related to Alzheimer’s disease, and superoxide dismutase 1 (SOD 1) involved in the onset of amyotrophic lateral sclerosis [4, 5]. In addition, dual specificity tyrosine phosphorylation-regulated kinase 1A (DYRK1A) and Down syndrome critical region gene 1 (DSCR1) on chromosome 21 are related to neurogenesis [6]. DYRK1A has attracted attention as a target for normalizing the phenotype of Down syndrome [7, 8]. DYRK1A inhibitor as a therapeutic agent for Down syndrome has been widely studied and developed and has been tested in clinical trials [9]. Additionally, low molecular weight molecules that improve the phenotype of Down syndrome have also been tested [10].

To develop drugs for Down syndrome, murine models for Down syndrome or trisomy 21 have been developed. Since the distal part of mouse chromosome 16 is orthologous to a large portion of human chromosome 21, mouse models, in particular the chromosome 16 segmental trisomies, Ts65Dn and Ts1Cje, were produced [11, 12]. These models are used for exploration of the etiology of Down syndrome and drug development [13–15]. Ts65Dn mice mimic the human condition, including developmental delay [16] and memory deficit, and may therefore be used for drug development with the aim of improving cognitive function [7]. Likewise, Ts1Cje carries a segmental trisomy of mouse chromosome 16 [12] and shows Down syndrome-related abnormalities such as craniofacial alterations [17] and spatial learning deficits [12]. Maternal supplementation of low molecular weight molecules such as epigallocatechin-3-gallate, fluoxetine, neuroprotective peptide, and choline during pregnancy improve function of these model mice [8, 10, 18, 19]. Neural stem cell-based therapy was also attempted with neonatal Down syndrome mice [20]. Further studies are necessary in order to determine the efficacy of these therapies.

Immortality of induced pluripotent stem cells (iPSCs) makes it possible to obtain a large number of cells from a small specimen, and pluripotency enables differentiation into various cell types [21–24]. Therefore, they are widely used to clarify disease etiology and test therapeutic drugs [25–28]. Attempts to normalize chromosomal abnormalities have been drawing intense research interest in the study of Down syndrome using iPSCs. In order to determine the mechanism of development of Down syndrome, normal cells are needed as controls. In a previous study, a comparison between monozygotic twins discordant for trisomy 21 had been performed [29]. Previous studies have reported normalization with using genome editing techniques and spontaneous correction during reprogramming to iPSCs [30–33]. In this study, iPSCs with the normal karyotype, i.e., chromosome 21-diploid cells, was detected at a high frequency in the process of culturing iPSCs derived from a patient with Down syndrome. In order to investigate the properties of trisomy 21 cells, we have characterized disomic and trisomic subclones that are isogenic with the exception of chromosome 21.

## Materials and methods

### Human cells

Amniotic fluid was obtained from a fetus with Down syndrome associated with polyhydroamnios. It was collected at 29 weeks of gestation for the purpose of reducing amniotic fluid. Cells were incubated in 4 mL of Amnio-MAX-II complete medium (Invitrogen, catalog number (#) 11269-016). Cell clusters appeared 6 to 7 days after seeding. Nonadherent cells were discarded and the medium replaced every 2 days. When the culture reached subconfluence, cells were harvested with a trypsin-EDTA solution (Wako, #209-16941) and re-plated at a 1:8 ratio in a 60-mm dish.

### Cell culture

Amniotic fluid-cells and iPSCs were cultured as described in previous literature [34–37]. iPSCs were maintained in E8 medium on VTN-coated dishes and passaged using 0.5 mM EDTA in PBS.

### Sub-cloning of iPSCs

Single cells were picked from colonies of iPSCs and cells were seeded at 1 cell/well in a 4-well plate coated with imatrix-511 (nippi, #892 012). Cells were cultured in StemFit AK02N (ReproCELL, #RCAK02N) supplemented with 10 μM Y-27632 (Wako, #251-00514) and those which showed colony formation were passaged.

### Real-time qPCR

RNA was extracted from cells using the RNeasy Mini kit (Qiagen, #74104). An aliquot of total RNA was reverse-transcribed using an oligo (dT) primer (Invitrogen, #18418-020). For the thermal cycle reactions, the cDNA template was amplified (Applied Biosystems Quantstudio 12 K Flex Real-Time PCR System) with gene-specific primer sets (Table 1) using the Platinum SYBR Green qPCR SuperMix-UDG with ROX (Invitrogen, #11733-046) under the following reaction conditions: 40 cycles of PCR (95 °C for 15 s and 60 °C for 1 min) after an initial denaturation (95 °C for 2 min). Fluorescence was monitored during every PCR cycle at the annealing step. mRNA levels were normalized using glyceraldehyde-3-phosphate dehydrogenase as a housekeeping gene.

**Table 1:** List of primers for qRT-PCR

Gene	Primer sequence	
DYRK1A	Forward	CTGGACTCTTCCCTCCCTTC
	Reverse	GCCGAACAGATGAAGGTTTG
APP	Forward	TTTGGCACTGCTCCTGCT
	Reverse	CCACAGAACATGGCAATCTG
SOD1	Forward	CTAGCGAGTTATGGCGACG
	Reverse	CCACACCTTCACTGGTCCAT
ETS2	Forward	GCCTCCCTGATCGTCTCTG
	Reverse	TGGTCCATATTCTTGATTCCG
DSCR1	Forward	AGTGGGATGGAAACAAGTGG
	Reverse	GCTGCGTGCAATTCATACTT
GAPDH	Forward	TGTTGCCATCAATGACCCCTT
	Reverse	CTCCACGACGTACTCAGCG
KRT14	Forward	GACCATTGAGGACCTGAGGA
	Reverse	CATACTTGGTGCGGAAGTCA
p63	Forward	GAAGATCCCATCACAGGAAGAC
	Reverse	GTTTCAATTGTGTGCTGAGGAA
TERT	Forward	GAGCAAGTTGCAAAGCATTG
	Reverse	TTTCTCTGCGGAAGGTTCTG
cycliD1	Forward	TGCTGCTGGAAATGCTGACT
	Reverse	TTTGTACAAGAAAGCTGGGT
CDK4R24C	Forward	TGCTGCTGGAAATGCTGACT
	Reverse	TTTGTACAAGAAAGCTGGGT

### Immunocytochemical analysis

Cells were fixed with 4% paraformaldehyde (PFA) in PBS for 10 min at 4 °C. After washing with PBS and treatment with 0.1% Triton X-100 (Sigma-Aldrich, #T8787-100 ML) for 10 min at 4 °C, the cells were incubated with 5% normal goat serum (Dako, #X 0907) in PBS for 30 min Pre-incubated at room temperature. Followed by reaction with primary antibody in blocking buffer for 24 h at 4 °C. After washing with PBS, the cells were incubated with fluorescently conjugated secondary antibody. Anti-rabbit or anti-mouse immunoglobulin G (IgG) bound to Alexa 488 or 546 (1:1000) was incubated in blocking buffer for 30 min at room temperature. The nuclei were stained with DAPI (Biotium, #40043). All images were captured using confocal microscopy (Confocal microscope C2+). Antibody information is provided in the Table 2.

**Table 2:** List of antibodies for immunochemistry

	Class	Company	Dilution
*Primary antibodies*			
Anti-PAX6 antibody	Rabbit IgG	abcam	1/350
Anti-Nestin antibody	Mouse IgG1	abcam	1/350
Keratin 14 polyclonal antibody	Rabbit polyclonal	BioLegend	1/1000
Anti-P63 (4A4) antibody	Mouse IgG2a	abcam	1/50
Monoclonal Anti-Involucrin antibody produced in mouse	Mouse IgG	Sigma-Aldrich	1/200
Anti laminin 5 antibody	Rabbit polyclonal	abcam	1/200
Loricrin polyclonal antibody	Rabbit polyclonal	BioLegend Inc	1/1000
Keratin 15 polyclonal antibody	Rabbit polyclonal	BioLegend Inc	1/1000
Anti-Ki67 antibody	Rabbit polyclonal	abcam	1/100
Anti-Pan-cytokeratin antibody	Mouse IgG1	eBioscience	1/200
*Secondary antibodies*			
Goat anti-rabbit IgG (H + L) Secondary antibody, Alexa Fluor 546	None	Invitrogen	1/1000
Goat anti-Mouse IgG1 Secondary antibody, Alexa Fluor 488	None	Invitrogen	1/1000
Goat anti-Mouse IgG3 Secondary antibody, Alexa Fluor 488	None	Invitrogen	1/1000
Goat anti-Mouse IgG2a Secondary Antibody, Alexa Fluor 488	None	Invitrogen	1/1000
Goat anti-Mouse IgG1 Secondary Antibody, Alexa Fluor 546	None	Invitrogen	1/1000
rabbit anti-Mouse IgG (H + L) Secondary Antibody, Alexa Fluor 488	None	Invitrogen	1/1000
Goat anti-rabbit IgG (H + L) Secondary antibody, Alexa Fluor 488	None	Invitrogen	1/1000

### Fluorescent in situ hybridization (FISH)

FISH analysis was performed using the ZytoLight FISH-Cytology Implementation Kit (Zytovision, #Z-2099-20). Potassium chloride solution 0.075 M was added to the trypsin-treated cell suspension and allowed to stand for 20 min, and then fixed with Carnoy solution. The Carnoy fixative was dripped onto the coverslip and air dried. Coverslips were immersed in 2 × SSC at 37 °C for 30 min. Cells were dehydrated in 70, 90, 100% ethanol for 2 m and air dried. Proteolysis and washing were then performed using the ZytoLight FISH-Cytology Implementation Kit according to the manufacturer’s protocol, followed by dehydration with 70, 90, and 100% ethanol for 1 min each, followed by air drying. After labeling the SPEC 21q22 probe (Zytolight, #Z-2086) according to the manufacturer’s protocol, the cells and probes were denatured on a hotplate at 72 °C for 2 min and hybridized overnight in a humidity chamber at 37 °C. Cells were then washed and mounted.

### Karyotypic analysis

Karyotypic analysis was performed at the Chromosome Science Labo Inc. Chromosome spreads were Giemsa banded and photographed. Twenty metaphase spreads were analyzed for each sample and karyotyped using a chromosome imaging analyzer system (Applied Spectral Imaging).

### Short tandem repeat analysis

STR analysis was conducted at BEX facility. Genomic DNA was used and 16 microsatellite markers were amplified by PCR using microsatellite specific primers.

### Growth curve

Trisomic or disomic iPSCs (1 × 10^5^ per well) were seeded in a 6-well plate coated with imatrix-511 (nippi, #892 012). The total number of cells/well was counted 2, 4, and 6 days after plating.

### Microarray analysis

RNA extraction and microarray analysis were performed at DNA Chip Research Inc. RNA extraction was performed using the Qiagen RNeasy mini kit (Qiagen, #74104) and cRNA synthesis was carried out according to the manufacturer’s protocol using Low Input Quick Amp Labeling Kit (Agilent, #5190-2305). Hybridization was performed using SurePrint G3 Human Gene Expression 8 × 60 K v3 (Agilent, G4858A #072363).

### Fluorescence-activated cell sorting analysis

The expression of cell-surface markers was analyzed by BD LSR Fortessa (BD Biosciences). Primary antibodies were incubated for 1 h in PBS with 1% BSA. After washing with PBS, cells were incubated with fluorescently coupled secondary antibodies; anti-rabbit IgG conjugated with Alexa 488 (1:1000) for 30 min at room temperature.

### Differentiation of iPSCs into neural stem cells (NSCs)

Differentiation of iPSCs into NSCs was accomplished using Neurobasal medium (Gibco, #21103049) according to the protocol. We subcultured iPSCs on VTN coated 60-mm dish in E8 medium on day 1. iPSCs were cultured in Neurobasal medium supplemented with neural induction supplement (Gibco, #A1647701) for 6 days. On day 7, the cells were passaged to 60-mm dish coated with geltrex (Gibco, #A1413202) and maintained in mixture of Neurobasal Medium and Advanced DMEM/F12 (Gibco, #12634010) (1:1) supplemented with neural induction supplement and 5 μM Y-27632.

### Differentiation of iPSCs into keratinocytes

The induction of differentiation into keratinocytes was carried out as previously described. We subcultured small clumps of undifferentiated iPSC on VTN coated 10-mm dish in E 8 medium on day 1. iPSCs were then cultured for 4 days in DKSFM (Invitrogen, #10744-019) supplemented with 1 mM all-trans RA (Wako, #182-01111) and 10 ng/mL bone morphogenetic protein 4 (BMP4) (R&D systems, #314-BP-010/CF). Subsequently, iPSC was maintained in DKSFM supplemented with 20 ng/mL EGF (R&Dsystems, #236-EG-200) for 10 days, then passaged to a 10-mm dish coated with 0.03 mg/mL type I collagen and 0.01 mg/mL fibronectin, and maintained in DKSFM supplemented with 10 μM Y-27632 (Wako, #251-00514) and 20 ng/mL EGF.

### Viral vector construction and viral transduction

Construction of the lentiviral vector plasmids CSII-CMV-Tet-Off, CSII-TRE-Tight-cyclin D1, and CSII-TRE-Tight-CDK4R24C was previously described [38]. In brief, the EF1a promoter in CSII-EF-RfA (a gift from Dr. H. Miyoshi, RIKEN) was replaced with a tetracycline-inducible promoter, TRE-Tight, from pTRE-Tight (Clontech, #631059) to generate CSII-TRE-Tight-RfA. Human cyclin D1, human mutant CDK4 (CDK4R24C: an INK4a-resistant form of CDK4), and hTERT were inserted into the entry vector via a BP reaction (Invitrogen, Carlsbad, CA). These segments were then recombined with CSII-TRE-Tight-RfA through an LR reaction (Invitrogen) to generate CSII-TRE-Tight-cyclin D1, CSII-TRE-Tight-CDK4R24C, and CSII-TRE-Tight-hTERT. The rtTA segment from pTet-Off Advanced (Clontech) was amplified by PCR, recombined with the donor vector pDONR221 via a BP reaction (Invitrogen) to generate pENTR221-Tet-Off, and then recombined with a lentiviral vector, CSII-CMV-RfA, through an LR reaction (Invitrogen) to generate CSII-CMV-Tet-Off. Recombinant lentiviruses with vesicular stomatitis virus G glycoprotein were produced as described previously [39]. Keratinocytes were inoculated with 5 × 10^6^ infectious units [IU] each of CSII-CMV-hTERT, CSII-CMV-Tet-Off, CSII-TRE-Tight-cyclin D1 and CSII-TRE-Tight-CDK4R24C lentiviruses in the presence of 4 μg/mL of polybrene.

## Results

### Reversion of chromosome 21 trisomy to disomy

**Fig. 1:**
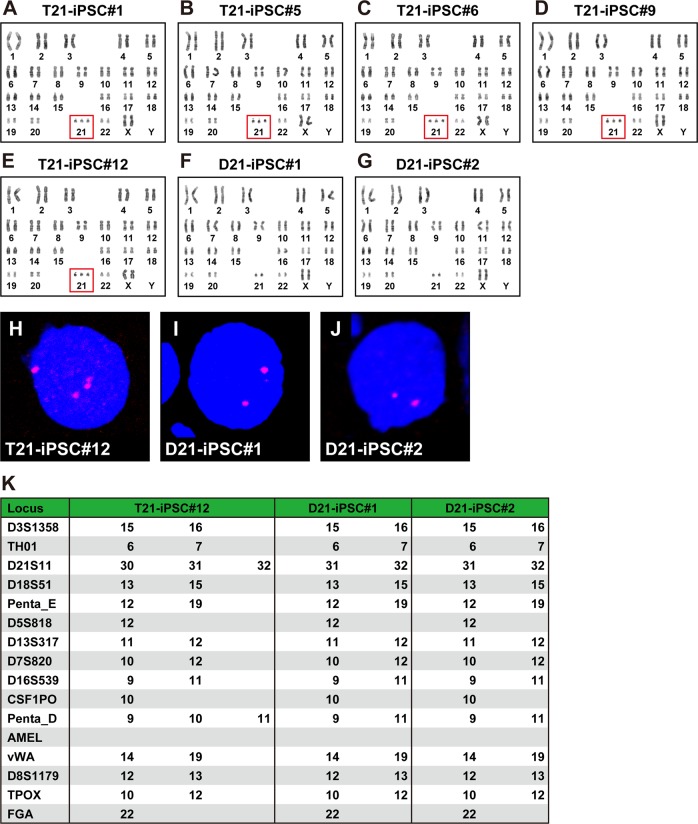
Trisomy rescue. **a** Karyotypic analysis in T21-iPSC #1. All of the cells (20 out of 20 cells) had typical trisomy karyotypes (47, XX, +21). **b** Karyotypic analysis in T21-iPSC #5. All of the cells (20 out of 20 cells) had typical trisomy karyotypes (47, XX, +21). **c** Karyotypic analysis in T21-iPSC #6. All of the cells (20 out of 20 cells) had typical trisomy karyotypes (47, XX, +21). **d** Karyotypic analysis in T21-iPSC #9. All of the cells (20 out of 20 cells) had typical trisomy karyotypes (47, XX, +21). **e** Karyotypic analysis in T21-iPSC #12. All of the cells (20 out of 20 cells) had typical trisomy karyotypes (47, XX, +21). This panel is duplicated from the reference 34. **f** Karyotypic analysis in D21-iPSC#1. D21-iPSC#1 had normal karyotypes (46, XX). **g** Karyotypic analysis in D21-iPSC#2. D21-iPSC#2 had normal karyotypes (46, XX). **h** FISH analysis in T21-iPSC#12 that was trisomic for chromosome 21. **i** FISH analysis in D21-iPSC#1 that was disomic for chromosome 21. **j** FISH analysis in D21-iPSC#2 that was disomic for chromosome 21. **k** STR analysis of T21-iPSC#12, D21-iPSC#1 and D21-iPSC#2

We established five independent iPSC lines (#1, #5, #6, #9, and #12) from amniotic fluid-derived cells from patients with Down syndrome. Karyotypic analysis revealed that the all iPSC #1, #5, #6, #9, and #12 exhibited chromosome 21 trisomy (47, XX, +21) in 100% of the lines (20 out of 20 metaphase cells) (Fig. 1a–e). We have been cultivating the iPSCs for more than 70 weeks (each passage was performed every 4–5 days). We have continuously investigated karyotypes of the iPSCs and observed normal chromosome 21 diploids (46, XX) in four out of 20 cells at passage 70 (corresponding to more than 200 population doublings). We subcloned two lines that were normal diploids from iPSC#12 with the single-cell dilution method (Fig. 1f, g). FISH analysis confirmed that the clones with chromosome 21 trisomy and disomy in the karyotypic analysis were indeed trisomic and disomic, respectively, for chromosome 21 (Fig. 1h–j). Trisomy 21 iPSCs and normal diploid iPSCs were designated as T21-iPSC#12 and D21-iPSC#2, respectively.

STR analysis was performed on these two D21-iPSC clones and T21-iPSC#12 to eliminate a possibility of contamination with other iPSCs (Fig. 1k). T21-iPSC#12 had three polymorphisms at two loci (D21S11 and Penta_D) on chromosome 21. In contrast, two D21-iPSC clones lost one repeat polymorphism in the two loci. Loss of the polymorphic pattern in D21S11 and Penta_D was the same in D21-iPSC#1 and D21-iPSC#2. The other STR patterns showed the same in T21-iPSC#12, D21-iPSC#1, and D21-iPSC#2, indicating that D21-iPSCs originate from T21-iPSCs.

### Comparison of T21-iPSCs and D21-iPSCs

**Fig. 2:**
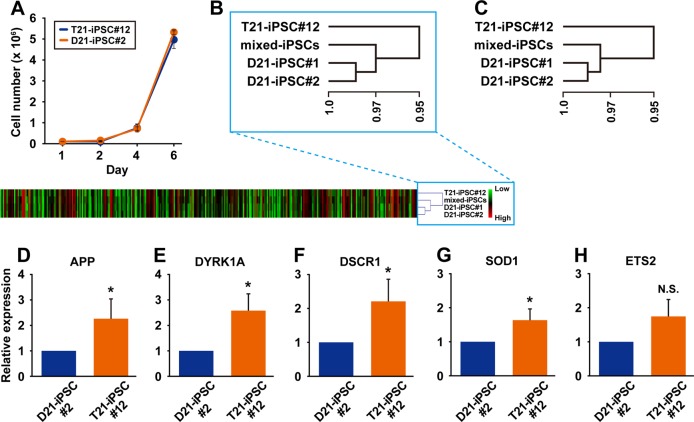
Comparison of trisomic and disomic iPS cells. **a** Growth rate of T21-iPSC#12 and D21-iPSC#2. Data shown are mean ± SD of the cell number from three independent experiments. **b** Heat map and hierarchical clustering of the normalized gene expression values in T21-iPSC#12, D21-iPSC#1, D21-iPSC#2, and mixed-iPSCs for the genes on chromosome 21. Hierarchical clustering of genes using Multi-Experiment Viewer. **c** Hierarchical clustering of the normalized gene expression values in T21-iPSC#12, D21-iPSC#1, D21-iPSC#2, and mixed-iPSCs for all the genes. Hierarchical clustering of genes using Multi-Experiment Viewer. **d** Quantitative RT-PCR analysis for expression of APP in T21-iPSC#12 and D21-iPSC#2. Data shown are mean ± SD of the expression from three independent experiments. *p < 0.05. **e** Quantitative RT-PCR analysis for expression of DYRK1A in T21-iPSC#12 and D21-iPSC#2. Data shown are mean ± SD of the expression from three independent experiments. *p < 0.05. **f** Quantitative RT-PCR analysis for expression of DSCR1 in T21-iPSC#12 and D21-iPSC#2. Data shown are mean ± SD of the expression from three independent experiments. *p < 0.05. **g** Quantitative RT-PCR analysis for expression of SOD1 in T21-iPSC#12 and D21-iPSC#2. Data shown are mean ± SD of the expression from three independent experiments. *p < 0.05. **h** Quantitative RT-PCR analysis for expression of ETS2 in T21-iPSC#12 and D21-iPSC#2. Data shown are mean ± SD of the expression from three independent experiments. N.S., not significant

We examined T21-iPSC#12 and D21-iPSC#2 to investigate difference of the growth rate. The growth rates of these two clones were comparable (Fig. 2a). We performed gene chip analysis on T21-iPSC#12, D21-iPSC#1, and D21-iPSC#2 to investigate gene expression levels. Expression levels of genes on chromosome 21 and all chromosomes are shown in heat map and hierarchical clustering analysis (Fig. 2b, c). D21-iPSC#1 and −2, i.e., two independent iPSC subclones with normal chromosome 21, are categorized into the same group. Up-regulated and down-regulated genes at 10-fold difference are listed in Tables 3 and 4. We then performed qRT-PCR analysis of the genes on Chromosome 21, based on the results of the gene chip analysis (Fig. 2d–h). The expression levels of the genes for APP (Alzheimer’s disease marker), DYRK1A, DSCR1 (Down-syndrome critical region 1), ETS2 and SOD1, all of which are located in chromosome 21, decreased to two-thirds in D21-iPSC#2, compared to T21-iPSC#12, implying that the revertant cells regained the gene expression levels of intact iPSCs.

**Table 3:** Up-regulated genes

Gene symbol	T21-iPSCs	D21-iPSCs-1	D21-iPSCs-2	Chromosome number	T21-iPSCs/D21-iPSCs-1	T21-iPSCs/D21-iPSCs-2
LEFTY1	358	19	24	chr1	19	15
FOXD3	493	46	151	chr1	11	3
RGS5	1177	59	194	chr1	20	6
lnc-ITGB3BP-1	1279	123	345	chr1	10	4
EPAS1	162	12	12	chr2	14	13
TTN	296	20	48	chr2	15	6
RAB17	2194	171	400	chr2	13	5
MME	151	15	45	chr3	10	3
TNIK	175	15	58	chr3	11	3
FLJ46120	286	21	59	chr3	14	5
KLKB1	190	17	48	chr4	11	4
AFP	387	32	55	chr4	12	7
SLC39A8	1067	97	151	chr4	11	7
CXCL14	384	25	43	chr5	15	9
lnc-MYO6-2	117	9	30	chr6	13	4
TRDN	1022	37	33	chr6	27	31
lnc-MACC1-1	110	13	9	chr7	9	13
lnc-MACC1-1	498	13	14	chr7	37	36
LOC101927668	1606	17	20	chr7	95	79
LOC101927668	1613	17	22	chr7	96	72
LOC101927668	1705	17	25	chr7	99	68
	540	16	31	chr8	34	18
	1204	38	45	chr8	32	27
ANXA1	193	22	19	chr9	9	10
XLOC_l2_002441	258	19	44	chr11	14	6
LOC100131262	465	45	86	chr11	10	5
GAL	13527	1142	1947	chr11	12	7
GDF3	241	9	16	chr12	27	15
METTL7A	694	50	87	chr12	14	8
NTS	704	56	55	chr12	13	13
LCP1	165	13	17	chr13	13	9
LHFP	953	79	173	chr13	12	5
lnc-MDGA2-2	1082	61	60	chr14	18	18
ZDHHC22	815	72	122	chr14	11	7
WFDC21P	122	10	15	chr17	12	8
MYH2	187	15	17	chr17	13	11
LINC01540	99	9	13	chr18	11	8
VSTM1	137	9	9	chr19	15	15
SEMG1	313	12	54	chr20	25	6
B3GALT5-AS1	100	9	22	chr21	11	5
	500	19	38	chr21	27	13
D21S2088E	2044	145	468	chr21	14	4
LOC100126447	896	69	132	chrX	13	7

**Table 4:** Down-regulated genes

Gene symbol	T21-iPSCs	D21-iPSCs-1	D21-iPSCs-2	Chromosome number	T21-iPSCs/D21-iPSCs-1	T21-iPSCs/D21-iPSCs-2
TXNIP	270	6137	4096	chr1	0.04	0.07
G0S2	54	209	1216	chr1	0.26	0.04
LOC100130502	33	2060	883	chr2	0.02	0.04
LOC440910	13	201	227	chr2	0.06	0.06
EPHA4	188	2820	1750	chr2	0.07	0.11
LOC100130502	33	449	291	chr2	0.07	0.11
	16	192	105	chr2	0.08	0.15
POTEI	140	1586	1610	chr2	0.09	0.09
lnc-SLC4A1AP-1	24	210	494	chr2	0.11	0.05
LIX1	10	551	237	chr5	0.01	0.02
ARRDC3	29	345	305	chr5	0.03	0.07
C6orf141	33	341	248	chr6	0.02	0.04
FEZF1-AS1	19	1341	609	chr7	0.04	0.08
FEZF1	12	222	112	chr7	0.07	0.10
LHX2	75	2111	784	chr9	0.14	0.08
LOC440896	12	143	86	chr9	0.02	0.05
PAX6	9	1061	488	chr11	0.03	0.09
LMO1	53	1984	731	chr11	0.04	0.09
LHX5-AS1	76	4092	2023	chr12	0.06	0.06
LHX5-AS1	12	330	148	chr12	0.08	0.09
VWF	38	570	392	chr12	0.08	0.13
DDIT3	26	180	337	chr12	0.13	0.06
DLK1	435	21476	8833	chr14	0.26	0.07
MEG3	22	711	237	chr14	0.23	0.07
DLK1	83	2319	901	chr14	0.24	0.08
POTEB3	57	1001	965	chr15	0.55	0.08
ARRDC4	112	1416	1281	chr15	0.87	0.09
PRTG	38	479	283	chr15	0.08	0.08
CPLX3	12	97	214	chr15	0.09	0.20
NUPR1	19	74	272	chr16	0.09	0.11
MT1M	108	477	1461	chr16	0.17	0.04
MT1E	240	988	3071	chr16	0.01	0.02
MT1G	254	466	3076	chr16	0.05	0.11
MT1H	162	186	1772	chr16	0.11	0.07
ARSG	11	133	136	chr17	0.09	0.12
BAHCC1	9	101	43	chr17	0.07	0.07
FLJ11710	51	549	464	chr17	0.09	0.09
TAC4	8	49	196	chr17	0.02	0.04
RAX	11	1387	588	chr18	0.08	0.09
RAX	16	337	152	chr18	0.10	0.13
APC2	61	564	907	chr19	0.01	0.03
GPCPD1	117	1238	1002	chr20	0.05	0.10
POTED	59	884	847	chr21	0.04	0.10
POTED	58	619	634	chr21	0.08	0.14
PNCK	10	108	79	chrX	0.10	0.13
	11	58	133	chrX	0.20	0.08
COPG2IT1	19	365	91		0.05	0.20

### Induction of differentiation into NSCs

**Fig. 3:**
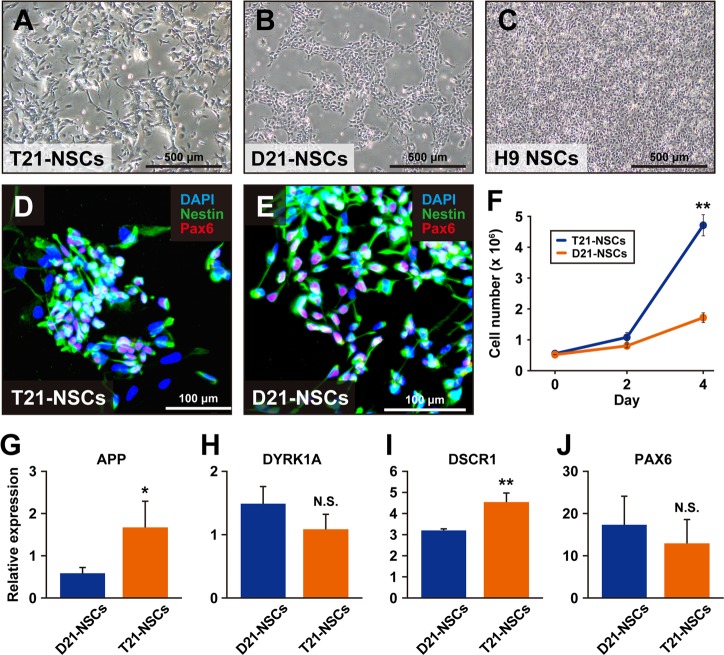
Generation of neural stem cells from iPSCs. **a** Microscopic analysis of neural stem cells derived from T21-iPSC#12 (T21-NSCs) at passage 4. **b** Microscopic analysis of neural stem cells derived from D21-iPSC#2 (D21-NSCs) at passage 4. **c** Microscopic analysis of human neural stem cells (H9-NSC). **d** Immunocytochemistry of T21-NSCs, using the antibodies to neural stem cell markers that are PAX6 (red) and Nestin (Green). Nuclei were counterstained with DAPI. **e** Immunocytochemistry of D21-NSCs, using the antibodies to neural stem cell markers that are PAX6 (red) and Nestin (Green). Nuclei were counterstained with DAPI. **f** The growth rate of T21-NSCs and D21-NSCs at passage 6. Data shown are mean ± SD of the cell number from three independent experiments. **p < 0.01. **g** Real-time qPCR analysis of APP. Data shown are mean ± SD of the expression from three independent experiments. *p < 0.05. **h** Real-time qPCR analysis of DYRK1A. Data shown are mean ± SD of the expression from three independent experiments. N.S., not significant. **i** Real-time qPCR analysis of DSCR1. Data shown are mean ± SD of the expression from three independent experiments. **p < 0.01. **j** Real-time qPCR analysis of PAX6. Data shown are mean ± SD of the expression from three independent experiments. N.S., not significant

We examined T21-iPSC#12 and D21-iPSC#2 for neural differentiation because Down’s syndrome patients have intellectual disability. T21-iPSC#12 and D21-iPSC#2 efficiently differentiated into NSCs in morphology and neural marker expression (Fig. 3). NSCs derived from T21-iPSC#12 (T21-NSCs) and D21-iPSC#2 (D21-NSCs) showed NSC-like morphology at passage 2. T21-NSCs and D21-NSCs were expressed NSC markers, i.e., PAX6 and Nestin. We measured the proliferation of T21-NSCs and D21-NSCs at passage 6. T21-NSCs grew more faster than D21-NSCs (Fig. 3f). qPCR analysis showed that the gene expression levels of APP and DSCR1 in T21-NSCs were higher than D21-NSCs (Fig. 3g, i). These results may suggest a possible link between clinical features of Down syndrome patients and T21-iPSC phenotypes.

### Keratinocytic differentiation of T21-iPSC and D21-iPSC

**Fig. 4:**
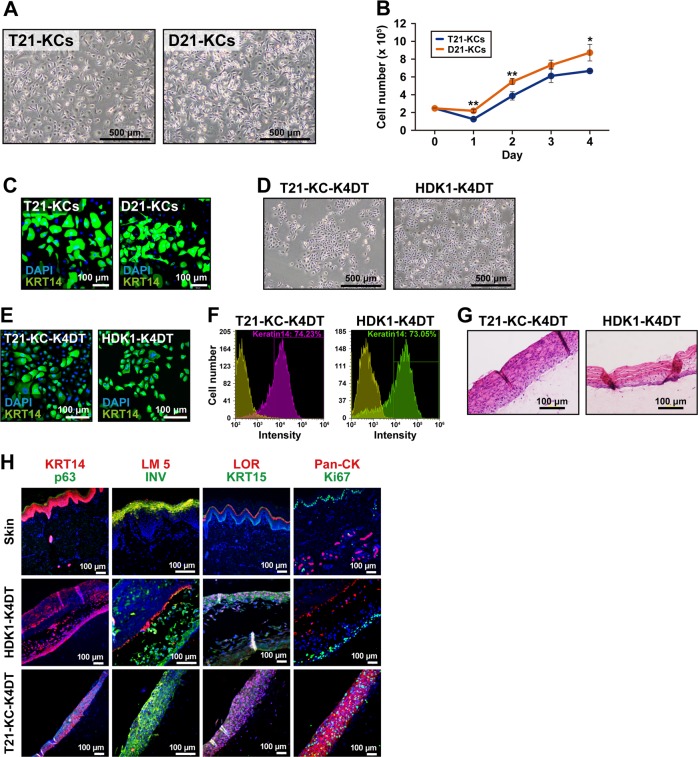
Generation of keratinocytes from iPSCs. **a** Microscopic analysis of keratinocytes derived from T21-iPSC#12 (T21-KCs) and D21-iPSC#2 (D21-KCs) at passage 2. **b** Growth rate of T21-KCs and D21-KCs at passage 2. Data shown are mean ± SD of the cell number from three independent experiments. **c** Immunocytochemistry of T21-KCs and D21-KCs with the anti-KRT14 antibody. Nuclei were counterstained with DAPI. **d** Phase-contrast photomicrographs of T21-KC-K4DT and HDK1-K4DT cells. **e** Immunocytochemistry of T21-KC-K4DT and HDK1-K4DT cells with the anti-KRT14 antibody. Counterstained with DAPI. **f** Flow cytometric analysis of T21-KC-K4DT and HDK1-K4DT cells with the anti-KRT14 antibody. Isotype controls are shown in each panel. **g** Histology of T21-KC-K4DT and HDK1-K4DT epidermis in 3D culture. HE stain. **h** Immunohistochemistry of intact skin, HDK1-K4DT epidermis and T21-KC-K4DT epidermis (from top to bottom) with the antibodies to KRT14, p63, LM5, INV, LOR, KRT15, Pan-CK and Ki67. Counterstained with DAPI

We generated iPSC-derived keratinocytes, based on a previously described protocol [34]. Keratinocytes were derived from T21-iPSC#12 (T21-KCs) and D21-iPSC#2 (D21-KCs) showed keratinocyte-like morphology at passage 2 (Fig. 4a). T21-KCs showed a slow growth rate compared with D21-KCs (Fig. 4b). Immunostaining revealed the expression of KRT14 in both T21-KCs and D21-KCs (Fig. 4c). Moreover, we immortalized T21-KCs to secure a stable supply and established 3-dimensional cultures for skin models. T21-KCs continued to proliferate in vitro and were infected with lentivirus carrying the CDK4R24C, cyclin D1, and hTERT genes. The immortalized iPSC-derived keratinocytes exhibited similar morphology to immortalized human keratinocytes (HDK1-K4DT) [40] (Fig. 4d). HDK1-K4DT was used for positive controls of immunocytochemistry or references. The infected cells were designated as T21-KC-K4DT. Immunocytochemistry and flow cytometric analysis clearly showed that T21-KC-K4DT cells were positive for KRT14 (Fig. 4e, f). T21-KC-K4DT formed stratified epithelium with keratinization after 3-dimensional cultivation (Fig. 4g). The T21-KC-K4DT epidermis expressed KRT14, p63, LM5, INV, LOR, KRT15, and Ki67 in a similar orientation to intact epidermis and HDK1-K4DT epidermis (Fig. 4h).

## Discussion

In this study, we introduced a spontaneous trisomy rescue in Down syndrome-derived iPSCs. Chromosome 21 trisomy has been reported to be mostly due chromosomal non-disjunction during meiosis I in the maternal egg. In contrast, paternal chromosomal non-disjunction occurs during meiosis II (spermatidogenesis). Non-disjunction of chromosomes during meiosis I and meiosis II result in heterologous pair of chromosomes and duplicated homologous pair of chromosomes, respectively. Presence of three different STR patterns in T21-iPSC#12 generated in this study suggests chromosomal non-disjunction during meiosis I. Possibility of other cell contamination can also be excluded by the same STR pattern between the trisomic and disomic cells. Furthermore, mosaicism of trisomic and disomic cells from the donor can also be excluded because the amniotic fluid-derived parental cells exhibit chromosome 21 trisomy exclusively and disomic cells have been reproducibly obtained from 100% trisomy cells.

Proliferation rates of trisomic cells are considered to be tissue-dependent; the proliferation rate of trisomic cells is low in mouse embryonic fibroblasts and in fibroblasts from Down syndrome fetuses, but high in hematopoietic cells [41–44]. Trisomy 21 cells are highly responsive to a variety of external stimuli acting through cell-surface receptors, such as genes encoding the interferon-α/β receptor and IFN-γ receptor on chromosome 21 [44]. The differential growth rates of iPSC-derived keratinocytes and NSCs in this study were consistent with this tissue-dependent Trisomy 21 cell growth.

Trisomy rescue arises from mitotic or meiotic nondisjunction, and the nondisjunction of chromosome 21 occurs more often in trisomic cells than in normal cells [45, 46]. Likewise, trisomic rescue during iPSC cultivation in this study can possibly be attributed to chromosomal nondisjunction. Interestingly, the proportion of trisomic cells in blood cells is lower than that in skin fibroblasts cultured from the same individual, and the frequency of trisomic rescue is tissue-dependent [44]. Elucidating the conditions in which aneuploid iPSCs produce revertant cells may facilitate the development of treatments for various chromosomal abnormalities.

Differences between trisomic and disomic cells are of interest because the genetic background of these cells is the same. The benefit of Down syndrome iPSC availability is pluripotency and immortality. Additionally, Down syndrome iPSCs can efficiently differentiate into neural and hematopoietic cells that are associated with mental retardation and leukemogenesis, respectively. Expression levels of Down syndrome-related genes such as SOD1, DYRK1A, ETS2, APP, and DSCR1 in chromosome 21 are comparable with the gene number, i.e., three 21 chromosomes. Cells rescued from trisomy serve as a good control for Down syndrome cells due to the same/similar genetic background. These differentiated cells can contribute to drug development for Down syndrome in two ways: (1) Correction of gene expression levels, i.e., ets2, dyrk1a, dscr1, and app; (2) Increased frequency of trisomic rescue. Drug re-profiling is most practical to fetus with Down syndrome as well as Down syndrome patients. Predicted usage may include treatment of mental retardation and prevention of leukemogenesis. Low molecular weight molecules have been clinically investigated in Down syndrome patients [9]. However, no studies have yet reported correction of mental retardation. Neurogenesis continues in the ventricular and subventricular zones of the cerebral cortex in the third trimester of pregnancy [47], and therapeutic intervention in fetus could therefore be possible. Drug may restore neurogenesis, enhance cortical growth, and improve the neurodevelopmental outcome of Down syndrome.
